# Breast Cancer Environment Centers and Advocacy: Baralt and McCormick Respond

**DOI:** 10.1289/ehp.1103466R

**Published:** 2011-05

**Authors:** Lori B. Baralt, Sabrina McCormick

**Affiliations:** Department of Women’s, Gender and Sexuality Studies, California State University, Long Beach, Long Beach, California, E-mail: lbaralt@csulb.edu; School of Public Health and Health Sciences, George Washington University, Washington, DC

Our findings regarding the Breast Cancer and Environment Research Centers (BCERCs) in which Wolff and Barlow are involved represent a broad overview of all four centers and are meant to portray several dimensions of the collaborative aspects of the work. In our article (Baralt and McCormick 2010), we aimed to advance the types of community-based participatory research projects exemplified by these centers. With this aim, we presented an analysis of the collaborative process to understand ways in which future funding can be better specified in the area of breast cancer and the environment. We sought to clarify how agencies can facilitate deepened participation in examining the potential underlying issues that may affect participatory research projects, particularly with regard to a lack of understanding of and training in community- based participatory research on the part of many scientists and advocates, as well as potentially divergent priorities or desired outcomes regarding the research.

The findings reported in our article (Baralt and McCormick 2010) show a need to further articulate participatory methods. We sought to make it clear throughout the article that our analysis provides a unique contribution to the dialogue about improving the collaborative process of participatory research projects. To this end, in a supplement to the article we provided recommendations that elaborated on the need for participants’ commitment to a participatory research approach, participatory research training for advocates and scientists, clearly defined roles for advocates in research, clearly defined decision-making processes, and deliberation and agreement on the allocation of funds. These recommendations were based on our findings and what we heard from both advocates and scientists when we asked them about how the process could be improved upon in the future.

Our analysis (Baralt and McCormick 2010) does not represent our review of the scientific merits of the research being done in the centers or the entirety of environmental breast cancer research, which has been in existence for many decades. The environmental breast cancer research to date has been of critical importance to science, policy making, and advocates who have also played an important role in advancing environmental breast cancer research beginning in the 1990s. The rigorous National Institutes of Health review processes necessary for each center to be funded assured that the BCERCs are innovative and compelling.

Our research (Baralt and McCormick 2010) provided a useful overview perspective on the collaborative process within the BCERCs, highlighting the strengths of the Bay Area BCERC, with the goal of improving similar projects in the future. We did not conduct ethnographic research in each center, which would have demonstrated more about the specific nature of collaboration in each location, as we noted that Van Olphen et al. (2009) nicely did with the Bay Area BCERC COTC. Rather, we provided an overview of the collaborative process (not the outcomes of the scientific research or COTC translation and dissemination activities) by assessing the perceptions of the collaboration by advocates and scientists who responded to our survey and were interviewed. This overview of the centers demonstrated, as noted by Wolff and Barlow, that the centers varied with regard to their experience with community-based participatory research. Wolff and Barlow note in their letter that the Bay Area COTC was the one center that incorporated the principles of community-based research and, based on our research, provided the best example of successful advocate–scientist collaboration among the BCERCs. Therefore, throughout our article (Baralt and McCormick 2010) we used the Bay Area BCERC (as well as in the Supplemental Material, in which we elaborated on a number of recommendations for future breast cancer–environment research collaborations) as a model for future collaborative projects. Additionally, we were careful in noting the limitations of our methods, acknowledging that our findings reflect only our sample of possible respondents and therefore may not be generalizable to all center advocates and scientists.

Our article (Baralt and McCormick 2010) and recommendations are both in the spirit of furthering the work of the BCERCs and projects like the BCERCs that engage in the “ongoing, interactive, collaborative, critical process of science and advocacy,” as Wolff and Barlow describe their work with the BCERCs over the past 7 years. We encourage other researchers to continue investigating how environmental breast cancer research and other types of participatory projects can best serve the interests of science, advocates and policy-makers.
